# Editorial: Fight against food waste: tackling pre-harvest diseases

**DOI:** 10.3389/fmicb.2023.1305400

**Published:** 2023-11-09

**Authors:** Yasmeen Siddiqui, Ajay Kumar, Ashish Srivastava

**Affiliations:** ^1^Department of Biological Sciences, College of Science, King Faisal University, Al Hofuf, Saudi Arabia; ^2^Amity Institute of Biotechnology, Amity University, Noida, India; ^3^Amity Institute of Virology and Immunology, Amity University, Noida, Uttar Pradesh, India

**Keywords:** food waste, pre-harvest diseases, phytopathogens, biological control, nanoemulsions, sustainable management

In a world where millions suffer from hunger and malnutrition, the shocking reality is that nearly one-third of the total food produced is wasted. Although food wastage occurs at various stages of the supply chain, one often overlooked area where significant losses occur is pre-harvest diseases. Pre-harvest diseases have no boundaries and affect the crops in both developed and developing nations with consequences reverberating throughout the food supply chain. Farmers face tremendous challenges in trying to control these diseases, often resorting to excessive pesticide use, which not only harms the environment but also contributes to the development of pesticide-resistant pathogens.

Addressing pre-harvest diseases is a multifaceted endeavor that requires a holistic approach. Recent research has paved the way for innovative approaches to protecting crops without harming the environment or human health. In this editorial, we highlight six groundbreaking studies that shed light on eco-safe alternatives to conventional pesticides and offer promising strategies for managing diseases that affect crops.

Mosa et al. underscored the potential of *Nigella sativa*, commonly known as black cumin, to combat *Penicillium verrucosum* infection in maize seeds. This study aimed to develop oil-in-water nanoemulsion fungicides using cold-pressed *N. sativa* oil to assess their antifungal properties. The researchers investigated the impact of sonication time and surfactant type on mean droplet size, polydispersity index, and zeta potential, using dynamic light scattering measurements. The findings revealed that both sonication time and emulsifier type significantly influenced the stability of the nanoemulsions, resulting in small particle sizes (168.6 to 345.3 nm), acceptable PDI values between 0.181 and 0.353, and zeta potentials ranging from −27.24 to −48.82 millivolts. Notably, Tween 20 demonstrated superior stability compared with Tween 80 nanoemulsions. *In vitro* tests demonstrated that T80 and T20 nanoemulsions at 100% concentration completely inhibited *P. verrucosum* growth. All nanoemulsions significantly enhanced maize seed germination, and stimulated root and shoot growth compared with those not infected by *P. verrucosum* or fungicide-treated seeds. Additionally, nanoemulsion seed treatment led to a significant increase in antioxidant activities and protease enzymes.

In a similar vein, Dorjee et al. emphasized the efficiency of essential oil-grafted copper nanoparticles (EGC) against maize fungal pathogens, including *Bipolaris maydis, Rhizoctonia solani, Macrophomina phaseolina, Fusarium verticillioides*, and *Sclerotium rolfsii*. The EGC showed considerable suppression of these pathogens *in vitro* at a low dosage of 20 mg ml^−1^, with ED_50_ values ranging from 43 to 56 g ml^−1^. Moreover, the incidence of maydis leaf blight in maize crops was significantly decreased *in vivo* when EGC was used as a seed treatment in conjunction with foliar spray at dosages of 250 mg L^−1^ and 500 mg L^−1^. In addition, it resulted in better photosynthetic activity, increased shoot biomass, improved plant height, greater germination percentages, enhanced vigor index, and enhanced root system features. However, higher EGC concentrations (1,000 mg L^−1^) exhibited phytotoxic effects that reduced growth, biomass, and copper bioaccumulation, notably in foliar-sprayed maize leaves. The absorption and concentration of vital micronutrients like manganese and zinc were also found to be significantly impacted by EGC and copper nanoparticles at this concentration. These nanoemulsions have the potential to serve as effective protective coatings for stored maize seeds, safeguarding them against fungal infections and promoting improved seed quality and growth.

The bacterium *Brevibacillus*, specifically strain *Br. brevis* HNCS-1, has attracted interest as a biocontrol agent. The strain, isolated from tea garden soil, displayed antagonistic qualities toward five pathogens that cause disease in tea plants. Yang et al. sequenced and examined the *Br. brevis* HNCS-1 genome to gain a deeper understanding of the genetic underpinnings of its biocontrol mechanism. Using orthologous genes, a phylogenetic analysis was carried out on *Br. brevis* HNCS-1 and 17 other available *Br. brevis* strains. Notably, 3,742 core genes were shared across Br. brevis HNCS-1 and the other *Br. brevis* strains when the protein-encoding genes were compared. The discovery of a non-ribosomal peptide synthetase (NRPS) gene cluster, known as “edeine,” among these core genes was of great interest. The edeine NRPS gene cluster of the HNCS-1 strain closely mirrored the edeine NRPS gene cluster that had previously been found. Researchers used ultra-high-performance liquid chromatography-tandem mass spectrometry (UHPLC-MS/MS) analysis to discover two key antibacterial peptides, edeine B and edeine A, in the HNCS-1 strain in order to determine the bioactive metabolites produced by Br. brevis. These discoveries emphasize Br. brevis HNCS-1's potential for use in agricultural applications, particularly in the fight against diseases of the tea plant, by illuminating the genetic and biochemical pathways underlying the biocontrol capabilities of this organism.

Aflatoxins are highly toxic fungal metabolites that seriously threaten the safety of the food supply globally. In a recent study, Jesmin et al. investigated the possibility that the nonpathogenic estuarine bacteria *Vibrio gazogenes* (Vg) could prevent the manufacture of aflatoxin in molds that produce the toxin. This study used the well-known aflatoxin producer *Aspergillus flavus* to examine the mechanism underpinning Vg-dependent aflatoxin inhibition. According to their research, the red pigment Vg-prodigiosin was taken up by fungal hyphae and was responsible for the reduction of aflatoxin synthesis following Vg treatment. Another prodigiosin producer, *Serratia marcescens*, also strongly suppressed aflatoxin synthesis, whereas non-producers including *Escherichia coli, Staphylococcus aureus, Vibrio harveyi*, and *Vibrio fischeri* did not. This further supported the significance of prodigiosin in aflatoxin inhibition. Furthermore, the biosynthesis of aflatoxin was directly inhibited by pure prodigiosin. It was indicated that the fungus's uptake of Vg-prodigiosin was endocytosis-dependent since filipin and natamycin, two endocytosis inhibitors, significantly increased aflatoxin synthesis while reducing Vg-prodigiosin uptake. Additionally, both hyphal fusion and branching, which are endosome-dependent processes, were markedly diminished by Vg administration. These results lend credence to the idea that endocytosis-dependent uptake of Vg-prodigiosin mediates Vg-associated aflatoxin inhibition, which most likely interferes with normal endosomal operations within the aflatoxin-producing fungus.

Further, Sallam et al. isolated 20 endophytic bacteria in healthy tomato plants and tested them against the pathogenic fungal isolates causing early blight disease. The growth of the fungal pathogens was significantly inhibited by three strains: *Enterobacter cloacae* HS-6, *Pseudomonas gessardii* HS-5, and *Pseudomonas mediterranea* HS-4. The antagonistic effects of various concentrations of bacterial culture filtrates (20, 40, and 60%) on the development of pathogenic fungus *in vitro* were investigated further by the researchers. Notably, when exposed to the 60% concentration of bacterial culture filtrates, the lowest dry weights of pathogenic isolates were found. Additionally, the study discovered that all culture filtrates included phenolic chemicals, which indicated their potential significance in preventing fungal development. In *in vivo* tests, *E. cloacae* and its culture filtrate outperformed other treatments by exhibiting the least disease severity in tomato plants affected by early blight. It was suggested that 1.3 glucanase gene expression increased significantly in treated plants.

In another article, Greer et al. provided an overview of significant diseases affecting brassicas as well as their management techniques. Cultural practices are more preventative than curative, and no single method of management is successful against all diseases. Chemical controls have drawbacks, environmental risks, and problems with resistance brought on by pathogens. Therefore, the review underlined the significance of creating site-specific integrated pest management (IPM) techniques. The assessment pinpointed information gaps and offered suggestions for effectively managing these serious diseases in brassica plants. Overall, this article functions as a guide for minimizing food loss by eliminating pre-harvest illnesses in brassica crops.

In conclusion, fighting food waste is both morally and practically necessary. The above findings highlight significant advancements in crop disease control that are environmentally benign in a time when sustainable agriculture is of the utmost importance. Novel approaches to safeguarding the food supply by harnessing the force of nature while lowering the risks to the environment and human health posed by using chemical pesticides have been carried out. However, more comprehensive *in vivo* and larger-scale field studies are necessary to evaluate the efficacy in real time.

## Author contributions

YS: Validation, Writing—original draft, Writing—review & editing. AK: Writing—review & editing. AS: Writing—review & editing.

